# Evolution of the oxyhemoglobin dissociation curve in COVID-19 related ARDS patients

**DOI:** 10.3389/fphys.2024.1463775

**Published:** 2024-11-11

**Authors:** Charlotte Dalne, Patrick Biston, Michaël Piagnerelli

**Affiliations:** ^1^ Intensive Care. CHU-Charleroi Chimay, Université Libre de Bruxelles, Charleroi, Belgium; ^2^ Experimental Medicine Laboratory, ULB 222 Unit, CHU-Charleroi Chimay, Université Libre de Bruxelles, Montigny-le-Tilleul, Belgium

**Keywords:** COVID-19, red blood cell, hypoxemia, hemoglobin, oxygen

## Abstract

**Introduction:**

Severe hypoxemia is the leading cause of admission in intensive care (ICU) in patients with COVID-19 related acute respiratory distress syndrome (ARDS). In these patients, several studies reported a left shift of the oxyhemoglobin dissociation curve associated with a lower mortality. However, these results are conflicting, as these studies include few patients and often no control groups. Moreover, the calculation of P50, representing the PaO2 value at which 50% of hemoglobin is saturated, is not corrected for factors known to influence it (pH, PaCO2 or temperature). For all of these reasons, we compared the corrected P50 between ICU patients with severe COVID-19 related ARDS on mechanical ventilation or not, and ARDS from other causes. We investigated the evolution of the corrected P50 during the first 3 days of ICU and its relationship with ICU mortality.

**Methods and Patients:**

We retrospectively calculated the corrected P50 in three groups of patients: intubated and mechanically ventilated COVID-19 related ARDS, non-intubated COVID-19 related ARDS and intubated patients with ARDS due to other infectious causes. The corrected P50 was calculated, on the worst blood gas analysis on days 1 and 3 of ICU admission, by the formula of Hill but modified by Dash et al., controlled for pH, PaCO2 and temperature. We collected ICU mortality.

**Results:**

463 blood gas analysis at days 1 and 3 from 214 ICU COVID-19 related ARDS patients (114 with intubation and 100 without) and 35 ICU patients with ARDS from other causes were analyzed. All patients were severely hypoxemic: PaO2/FiO2 of 76 [58-108] mmHg for intubated COVID-19, 79 [60-108] mmHg for non-intubated COVID-19 and 142 [78-197] mmHg for the third group (*p* < 0.001). The mortality rate was higher in intubated COVID-19 related ARDS patients (44.7 versus 14 versus 37% in ARDS from other causes; *p* < 0.001). The corrected P50 was significantly lower in COVID-19 patients, especially in non- intubated patients (21.2 [18.8–25.2] mmHg vs. 25.5 [19.2–30.3] mmHg in intubated patients; compared to ARDS from other causes: 27.2 [23.3-35.4] mmHg; *p* < 0.001. The corrected P50 does not change over the first 3 days, except for the non intubated COVID-19 related ARDS and is not correlated with ICU mortality (odds ratio = 0.98 [0.95-1.03]; *p* = 0.51), in contrast of PaO2/FiO2 and ICU gravity scores.

**Conclusion:**

The oxyhemoglobin dissociation curve at ICU admission was left shifting in severe COVID-19 related ARDS patients regardless of the type of ventilation. This deviation increases the third day only in non-intubated COVID-19 related ARDS and was not related to the outcome.

## Introduction

Since the end of 2019, there have been more than 771 million cases of infection due to severe acute respiratory syndrome coronavirus 2 (SARS-CoV-2), and 6.9 million deaths (World Health Organization, 2023). In Belgium, the number of SARS-CoV-2 positive cases reached 4.8 million cases with 34,300 deaths (Sciensano, 2023). Patients admitted to the intensive care unit (ICU) represent 20%–25% of hospital admissions for COVID-19 with 70% of them requiring mechanical ventilation for acute respiratory distress syndrome (ARDS). Despite better knowledge of the disease and some potential therapies, the mortality rates for COVID-19 ARDS remain around 35%–40% worldwide (COVID-ICU group on behalf of the REVA Network and the COVID-ICU Investigators, 2021; Auld et al., 2022; Greco et al., 2022).

One of the characteristics reported in COVID-19-related ARDS is that patients clinically tolerate very severe hypoxemia well, unlike ARDS patients with other etiologies. This phenomenon has been called ‘silent or happy hypoxemia’ and the etiology of which remains controversial (Dhont et al., 2020). This clinical aspect of well tolerated hypoxemia would increase the risk of mortality in these patients, as less is paid to the need for non-invasive or invasive ventilation (Dhont et al., 2020). One of the hypothesis of this “silent hypoxemia” is a left shift of the oxygen hemoglobin dissociation curve (ODC). This modification facilitates oxygen (O2) uptake in damaged lungs, but reduces theoretically the capacity of hemoglobin to release O2 to the tissues. Despite this, the left shifting of ODC is associated with a reduction in mortality (Böning et al., 2023). Several etiologies are put forward: increase in metabolites of glycolysis observed in red blood cells (RBCs) from COVID-19 patients could suggest an increase in the capacity of hemoglobin to off-load O2 as a function of allosteric modulation by high energy phosphate compounds (Thomas T et al., 2020). Another explanation is the complex metabolism of intraerythrocytic 2,3 diphosphoglycerate (2,3 DPG). Two possible mechanisms could explain the decrease of intraerythrocytic concentrations: hyperventilation decrease alveolar carbon dioxide partial pressure (PaCO2) and increase RBC hydrogen potential (pH), and secondly: senescent circulating RBCs or RBCs with altered rheology sometimes observed in COVID-19 patients have a lower synthesis capacity of 2,3DPG. This later remains controversial, since unlike (Rogers et al., 2024), we (Piagnerelli M et al., 2022) and other (Favaron et al., 2021) did not found alterations in RBCs deformability and microcirculation in COVID-19 patients compared with septic patients.

Hemoglobin affinity for O2 is determined by its P50, which corresponds to the PaO2 at which 50% of hemoglobin molecules are saturated with O2. This P50 can be traced on the ODC, which is a sigmoid curve representing the saturation of hemoglobin in O2 at different PaO2 in the body.

Several parameters modify this curve, including PaO2, PaCO2, temperature, 2,3 DPG concentrations, pH, methemoglobin (metHb) and anemia (Vogel D et al., 2020). Recently, Böning D et al. reported a review of 14 studies investigating the P50 in COVID-19 patients. The results are heterogeneous probably due to the study methods (arterial and/or venous blood samplings, measurements at admission or during all the hospital stay.), the type of patients studied (few were severely ill ICU patients) and the control groups (ARDS from other causes, healthy controls) but also the calculation of the P50 without correction for S02, PaCO2, 2,3 DPG, pH and temperature (Böning D et al., 2023).

For these reasons, this study will compare corrected P50 and its evolution in three groups of severely ill ICU patients: intubated patients with severe COVID-19-related ARDS, non-intubated patients with severe COVID-19-related ARDS and patients with non-COVID- 19-related ARDS, as well as the possible association with ICU mortality.

## Methods

After approval by the ISPPC ethics committee on 05/12/22 (OM008; TFE22/46_30/11), which waived the patient’s informed consent in view of the retrospective nature, we included all COVID-19 patients and patients with non-COVID-19 related ARDS due admitted from March 2020 to February 2022 to the CHU-Charleroi Chimay hospital, Marie- Curie and Vesale sites. Data were already collected and anonymized in an Excel file. The patients included were divided into three distinct groups according to the following criteria: two groups concerned COVID-19 related ARDS proven by a positive Polymerase Chain Reaction (PCR) and hospitalized between March 2020 and March 2021. The first was intubated and mechanically ventilated and the second group concerned non-intubated patients with ARDS related to COVID-19. The third group concerns intubated and mechanical ventilated patients with sepsis or septic shock due to ARDS of other infectious etiologies and hospitalized between October 2020 and February 2022. ARDS was classified according to the Berlin definition (ARDS Definition Task Force et al., 2012). Sepsis is defined as life- threatening organ dysfunction caused by a dysregulated host response to infection. Septic shock is a subset of sepsis with profound circulatory (requiring norepinephrine to maintain a mean arterial pressure greater than 65 mmHg despite adequate fluid resuscitation), cellular and metabolic abnormalities (assess by a lactate level ≥ 2 mmol/L) (Singer et al., 2016). All patients admitted to the ICU with a blood gas at days 1 and 3 were included. All patients had to have the same type of ventilation (invasive or not) until day 3. We have excluded patients with a pulmonary embolism, pregnant women, patients with hemoglobin disease (sickle cell anemia, thalassemia, etc.), patients transfused during the study period and treated by nitric oxide (NO).

We collected demographic data, ICU gravity scores: APACHE II (Knaus et al., 1985) and sepsis-related organ failure assessment (SOFA) score (Vincent et al., 1996), biological data, length of ICU and hospital stay, ICU and hospital mortalities. The blood gases with the lowest PaO2/FiO2 ratio on D1 and D3 were reported and were used to calculate the corrected P50.

### P50

P50 depends on several parameters: temperature, pH, PaCO2, PaO2 and oxygen saturation (SO2). The Hill formula corrected by Dash et al. (Dash et al., 2016) was used to calculate P50. The Hill equation including the Hill coefficient is a widely used P50 equation but it presents errors for saturations below 30% and above 98% (Dash et al., 2016). This is why a coefficient has been added, which depends on PaO2 and allows saturation values from 0% to 100% to be used (Ceruti et al., 2022). The correction to the Hill equation made by Dash et al. was added to counteract the leftward shift of hypocapnic patients (Ceruti S et al., 2022). 2-3BPG was not measured in our database, so it was taken as a fixed value of 4.65 mmol/L, as described in the article by Ceruti S et al. (2022). The corrected P50 formula used is therefore as follows (Dash et al., 2016; Ceruti S et al., 2022):

P50 Formula

$${PO_{2}\times \left(\frac{1-SO_{2}}{SO_{2}}\right)}^{\frac{1}{2.82-1.2\times 10^{-\left(\frac{PO_{2}}{29.25}\right)}}}$$

P50 Formula corrected

$${P50}_{corrected}=P50\times \frac{p50,∆pH}{p50}\times \frac{p50,∆{CO}_{2}}{p50}\times \frac{p50,∆T}{p50}$$

where

$$\begin{array}{ll} P50,∆pH & =p50-25.535\times \left(pH-7.4\right)+10.646\times {\left(pH-7.4\right)}^{2}\\ & \;-1.764\times {\left(pH-7.4\right)}^{3} \end{array}$$

$$\begin{array}{ll} p50,∆{CO}_{2} & =p50+1.273\times 10^{-1}\times \left(p{co}_{2}-40\right)+1.083\times 10^{-4}\\ & \;\times {\left(p{CO}_{2}-40\right)}^{2} \end{array}$$

$$\begin{array}{ll} p50,∆T & =p50+1.435\times \left(T-37\right)+4.163\times 10^{-2}\times {\left(T-37\right)}^{2}\\ & \;+6.86\times 10^{-4}\times {\left(T-37\right)}^{3} \end{array}$$

It should also be noted that the reference value for P50 is 26.7 mmHg, based on the standard hemoglobin deviation curve for a PaCO2 of 40 mmHg, a pH of 7.4 and a temperature of 37°C in healthy volunteers (Aberman et al., 1973; Bergamaschi et al., 2023).

### Statistical analysis

Descriptive statistics were used to determine the median of the various demographic and biological data with the interquartile ranges [25%–75%]. The Kruskal–Wallis test was used to compare the median values of the demographic data, the respiratory parameters, the biological data and the P50 between the three groups. When the median values were statistically different, they were compared two by two using the Dunn test. The Wilcoxon test was used for dependent samples (data collected on day 1 and day 3).

Corrected P50 were compared between groups on day1 using the Mann-Whitney test, and Spearman’s correlation was used between corrected P50, SOFA and Apache II scores, age, lactate concentrations and PaO2/FiO2. Relationship between corrected P50 at day 1 and mortality was assessed by logistic regression. All statistical tests are considered significant for a *p*-value <0.05.

## Results

A total of 283 patients were included on day 1, but 34 patients have exclusion criteria (17 were order to not intubated, 11 were rapidly on vvECMO, three have a hematological disease, two have a PaO2/FiO2 > 300 and one was transfused during the study period). In the end, 249 patients were studied, divided into 114 intubated COVID-19 related ARDS patients, 100 non- intubated COVID-19 related ARDS patients and 35 non-COVID-19 ARDS patients with or without septic shock.

Table 1 shows the demographic data of the study population. The majority of patients were male, with a median age between 63 and 67 years and a BMI between 25.9 and 29.9 kg/m^2^. Pulmonary history, immunosuppression and BMI were statistically different between the groups. Almost 50% of non-COVID-19 ARDS patients had pre-existing lung disease, compared with only 20% of COVID-19 intubated patients. APACHE II score was significantly higher in the non-COVID-19 group and in intubated COVID-19 compared with non-intubated COVID-19. The non-intubated COVID-19 patients have a lower SOFA score than the other two groups (table 1). As expected, non-intubated COVID-19 patients have lower hemodynamic instability and were less inflammatory (Table 2). Hemoglobin concentrations were significantly lower in patients with ARDS due to other etiologies at day 1 and day 3 (Table 2).

**TABLE 1:** Baseline clinical characteristics and outcome according to patient groups.

	Intubated COVID-19 ARDS (n = 114)	Non-intubated COVID-19 ARDS (n = 100)	Non-COVID-19 ARDS (n = 35)	*p*-Value
Age (years)	67 [60-74]	63 [53-71]*	64 [59-75]	0.03
Female (%)	36 (32)	24 (24)	13 (37)	0.26
Cardiomyopathy (%)	23 (20)	22 (22)	13 (37)	0.11
Arterial hypertension (%)	67 (59)	46 (46)	21 (60)	0.13
Diabetes mellitus (%)	42 (37)	33 (33)	13 (37)	0.98
Lung diseases (%)	25 (22)	33 (33)	17 (49)*	0.008
Hepatic diseases (%)	9 (8)	8 (8)	2 (6)	0.9
Chronic renal failure (%)	17 (15)	14 (14)	5 (14)	0.93
Malignancy (%)	8 (7)	4 (4)	4 (12)	0.29
Immunocompromised (%)	7 (6)	14 (14)	7 (20)*	0.04
BMI (kg/m2)	29.5 [26.5-35.0]	29.9 [26.0-34.3]	25.9 [22.6-31.6]* £	0.008
APACHE 2 score	15 [11-19]	11 [9-15]*	20 [15-24]* £	<0.001
SOFA day 1	7 [4-8]	3 [2-4]* $	8 [5-11]	<0.001
SOFA day 3	6 [3-8]	2 [2-3]* $	7 [3-10]	<0.001
Length mechanical ventilation (days)	10 [6-16] $	0 [0-0]* $	3 [0-8] £	<0.001
ICU length of stay (days)	12 [8-19]	5 [4-8]*	8 [5-13]*	<0.001
Hospital length of stay (days)	19 [12-27]	14 [11-20]*	15 [9-29]	0.035
ICU mortality (%)	51 (45)	14 (14)*	13 (37)	<0.001
Hospital mortality (%)	53 (47)	19 (19)	16 (46)	0.31

BMI: Body Mass Index. APACHE II, score; ICU, gravity score. SOFA: sepsis-related organ.

failure assessment.

*p < 0.05 vs. intubated COVID-19 ARDS.

£*p* < 0.05 vs. non-intubated COVID-19 ARDS.

**TABLE 2:** Hemodynamic and biological characteristics of the patients.

	Intubated COVID-19 ARDS (n = 114)	Non -intubated COVID-19 ARDS (n = 100)	Non-COVID-19 ARDS (n = 35)	*p*-Value
Temperature day 1 (°C)	36.9 [36.3-37.7]	36.4 [36.0-37.0]*	36.6 [35.6-37.5]	0.005
Temperature day 3 (°C)	36.8 [36.3-37.6]	36.4 [36.0-36.8]*	36.7 [36.0-37.4]	<0.001
MAP day 1 (mmHg)	79 [68-93]	91 [81-99]* $	72 [64-89]	<0.001
MAP day 3 (mmHg)	81 [75-90]	92 [82-102] *	82 [73-102]	<0.001
HR day 1 (bpm)	85 [70-102]	83 [71-97]	103 [90-118]* £	<0.001
HR day 3 (bpm)	76 [65-87]	82 [69-95]	91 [81-111]*	<0.001
RR day 1 (cycles par minute)	25 [20-29]	24 [21-30]	24 [18-28]	0.46
RR day 3 (cycles par minute)	25 [21-30]	24 [20-29]	24 [20-29]	0.24
SaO2 day 1 (%)	93 [91-96]	93 [91-95]	94 [91-96]	0.43
Sa O2 day 3 (%)	93 [92-95]	94 [92-95]	94 [92-96]	0.47
Vasopressors day 1 (µg/kg min)	0.06 [0.0-0.15]	0.0 [0.0-0.0] * $	0.21 [0.0-0.30]	<0.001
Vasopressors day 3 (µg/kg min)	0.0 [0.0-0.05]	0.0 [0.0-0.0] * $	0.04 [0.0-0.33]	<0.001
Hemoglobin day 1 (g/L)	125 [113-135]	129 [118-140]	109 [90-129]* £	<0.001
Hemoglobin day 3 (g/dL)	116 [105-125]	126 [113-131]	101 [81-118]* £	<0.001
WBC day 1 (G/L)	8.8 [6.6-13.9]	8.0 [6.0-11.2]	14.5 [7.2-18.1]*	<0.001
WBC day 3 (G/L)	9.9 [7.8-13.1] £	8.7 [6.5-11.0]	12.4 [10.0-16.8]*	<0.001
C-Reactive Protein day 1 (mg/L)	174 [102-228]	99 [56-149]* $	220 [110-378]	<0.001
C-Reactive Protein day 3 (mg/L)	88 [57-169]	41 [23-78] * $	142 [74-308]	<0.001

*p < 0.05 vs. Intubated COVID-19 ARDS.

£*p* < 0.05 vs. non-intubated COVID-19 ARDS.

$p < 0.05 vs. non COVID -19 ARDS.

MAP: mean arterial pressure, HR: heart rate; RR: respiratory rate; SaO2, arterial O2 saturation, WBC: white blood count.

ICU mortality was 44.7% in intubated COVID-19 related ARDS, 37.1% in non-COVID-19 related ARDS and 14% in non-intubated COVID-19 related ARDS patients (Table 1).

### Blood gases analysis and P50

All the COVID-19 related ARDS were severe according to the Berlin classification (ARDS Definition Task Force et al., 2012) (Table 3). PaO2 was significantly higher in non-COVID-19 patients than in all COVID-19 patients without difference between groups in SaO2 (Table 3).

**TABLE 3:** blood gases analysis of the patients.

	Intubated COVID-19 ARDS (n = 114)	Non-intubated COVID-19 ARDS (n = 100)	Non -COVID-19 ARDS (n = 35)	p-Value
pH day 1	7.39 [7.31-7.44]	7.48 [7.45-7.49] * $	7.35 [7.22-7.42]	<0.001
pH day 3	7.41 [7.37-7.44]	7.47 [7.45-7.49] * $	7.42 [7.36-7.45]	<0.001
PaCO2 day 1 (mmHg)	42 [36-47]	34 [31-38] * $	39 [33-49]	<0.001
PaCO2 day 3 (mmHg)	44 [40-51]	35 [31-39] * $	43 [34-49]	<0.001
PaO2 day 1 (mmHg)	60 [52-70]	61 [56-68]	68 [62-78] * £	<0.001
PaO2 day 3 (mmHg)	61 [57-67]	62 [55-71]	73 [67-82] * £	<0.001
PaO2/FiO2 day 1 (mmHg)	76 [58-108]	79 [60-108]	142 [78-197]* £	<0.001
PaO2/FiO2 day 3 (mmHg)	104 [79-126]	88 [66-142]	190 [125-232] * £	<0.001
CaO2 day 1 (mLO2/dL)	15.60 [13.96-16.98]	15.80 [14.88-17.09]	14 .22 [11.95-16.99] £	0.021
CaO2 day 3 (mLO2/dL)	14.59 [12.90-15.62]	15.84 [14.30-16.79] * $	12.65 [11.00-14.76]*	<0.001
Lactate day 1 (mmol/L)	1.2 [1.0-1.6]	1.1 [0.9-1.5]	2.1 [1.0-3.4] * £	<0.001
Lactate day 3 (mmol/L)	1.4 [1.2-1.7]	1.3 [1.0-1.6]	1.3 [1.0-1.9]	0.23
Base Excess day 1 (mEq/L)	−0.1 [-3.3; 2.4]	1.0 [-0.6; 3.8]* $	−3.1 [-7.4; 0.7]	<0.001
Base Excess day 3 (mEq/L)	2.8 [-0.1; 5.1]	1.6 [-0.9; 3.5]	2.0 [-2.3; 6.0]	0.38
FIO2 day 1 (%)	80 [60-100]	80 [60-100]	60 [35-95] * £	<0.001
FiO2 day 3 (%)	60 [50-70]	78 [45-100]	40 [31-60]* £	<0.001

*p < 0.05 vs. Intubated COVID-19 ARDS.

£p < 0.05 vs. non-intubated COVID-19 ARDS.

$*p* < 0.05 vs. non COVID-19 ARDS.

CaO2 = (SaO2 x [Hemoglobin] x 1.34) + (PaO2 x 0.0031).

[Hemoglobin] was express in g/dL.

PaCO2 values were significantly lower and pH more elevated in non-intubated COVID-19 related ARDS (Table 3), without significant change in respiratory rate (Table 2). These modifications in PaCO2 persist at day 3.

**FIGURE 1:**
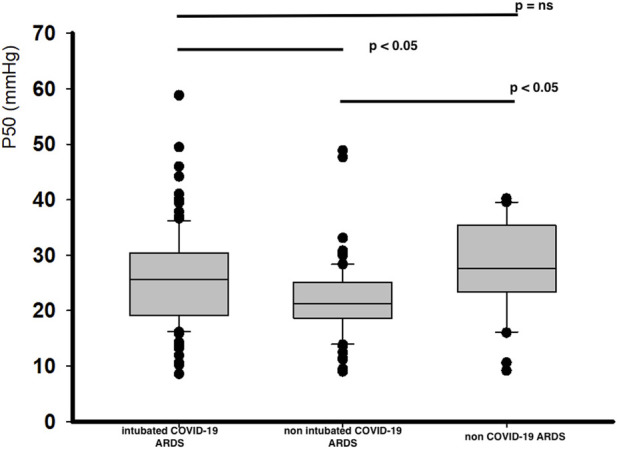
Corrected P50 at day 1 for all groups.

COVID-19 related ARDS patients have a significant lower P50 value at day 1, with the lowest for non-intubated COVID-19 related ARDS patients (Figure 1). These differences in corrected P50 between the groups remained marked at day 3 without significant modifications in P50 between day 1 and day 3, except for the non-intubated COVID-19 related ARDS (day 3 for intubated COVID-19 related ARDS: 24.5 [20.2-28.9] mmHg, *p* = 0.37; for non intubated COVID-19 related ARDS patients: 20.7 [15.5-23.1] mmHg, *p* = 0.02 and 27.2 [20.5-34.2] mmHg for non COVID-19 related ARDS, *p* = 0.79 compared with Day 1). No significant difference was observed at day 1 in relation to outcome for intubated COVID-19 related ARDS (alive: 25.5 [19.0-30.7] mmHg versus 26.3 [19.8-29.6] mmHg for deceased patients; *p* = 0.89) or for the evolution between day 1 and day 3 for each groups (for alive patients: day 1: 25.5 [19.0-30.7] mmHg versus day 3: 23.7 [19.6-28.0] mmHg, *p* = 0.15; and for deceased patients: day 1: 26.3 [19.8-29.6] mmHg versus day 3: 26.6 [21.1-31.3], *p* = 0.74).

**FIGURE 2:**
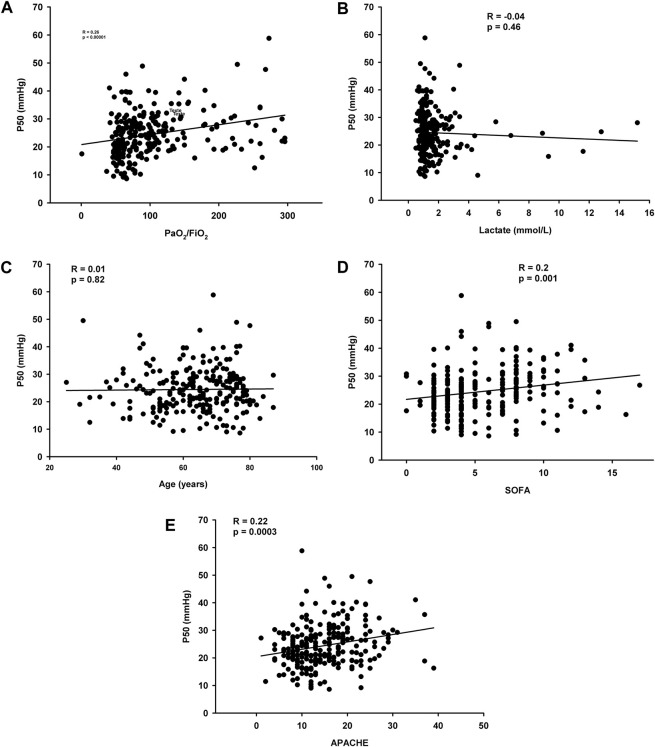
**(A)** Correlation between corrected P50 and PaO2/FiO2 **(B)** Correlations between corrected P50 and lactate concentrations **(C)** Correlations between corrected P50 and age **(D)** Correlations between corrected P50 and SOFA day 1 **(E)** Correlations between corrected P50 and APACHE II.

Logistic regression shows no relationship between corrected P50 at day 1 and ICU mortality (odds ratio = 0.98 IC [0.95-1.03], *p* = 0.51). Spearman’s correlation for the three groups combined suggests that corrected P50 is not significantly correlated with, lactate concentrations (r = −0.04, *p* = 0.46) and age (r = 0.01, *p* = 0.82) but with PaO2/FiO2 (r = 0.26, *p* < 0.0001) and ICU gravity scores (APACHE II: r = 0.22, *p* = 0.0003 and SOFA score at day 1: r = 0.20, *p* = 0.001 (Figure 2).

## Discussion

In this retrospective observational study, we observe at day 1 of the ICU admission a left shifting of the ODC in all COVID-19 related ARDS patients, especially in non-intubated patients, compared with intubated non-COVID-19 related ARDS patients. This left shifting persists at day 3 and was not associated with ICU mortality.

### Comparisons with other studies

#### Study population

Studies on ODC in COVID-19 related ARDS patients report conflicting results with left, right shift or no effect on the ODC (Böning et al., 2023). Vogel et al. (2020), Valle et al. (2022) and Ceruti et al. (2022) also reported a leftward shift of the ODC in COVID-19 patients. Nevertheless, there are many differences between these studies and our own, making it difficult to compare results. In the study of Vogel et al. (2020), they analyzed only in 43 intubated COVID-19 related ARDS patients the results of both arterial and venous blood gases over the complete ICU length of stay. These patients were both more anemic (mean hemoglobin concentrations: 81.2 ± 12.4 g/L) and less hypoxemic than our population (PaO2/FiO2: 215.7 ± 111.5 mmHg in COVID-19 related ARDS patients and 299.4 ± 236.5 mmHg in controls). These two factors may influence the results of the P50.

Ceruti et al. (2022) reported a mean value of all blood gases analysis in 32 intubated COVID-19 related ARDS patients with a minimum length of ICU stay of 7 days. They also observed a leftshift of the ODC and persistence of this leftshift was associated with a good outcome. In comparison with our results, patients had a higher PaCO2 and were also more anemic. In addition, no control group was included for comparison. After a comment on methodology by Böning et al. (2023), the authors recalculated the P50 and still observed this difference between surviving and deceased patients but with a significant right shift of the ODC after 7 days of ICU stay in patients with poor outcome, possibly suggesting a failure of respiratory compensation (Ceruti et al., 2022). Valle et al. (Valle et al., 2022) reported results on a great number of patients (517 COVID-19 and 314 control patients) but they calculated P50 only in one blood gases analysis before treatment and these patients were less severely ill than in our study.

The study by Daniel et al. (2020), which compared COVID-19 patients to healthy with the same demographic characteristics (age and sex), showed no difference in the ODC between both groups. Nevertheless, this study was carried out on only 14 COVID-19 patients (including 10 ICU intubated patients and four admitted to the emergency department) compared with 11 control patients.

In non ICU patients, Bergamaschi et al. like Ceruti et al. (Ceruti et al., 2022) reported a relationship between P50 and severity of hypoxemia. They observed a rightshift of the ODC in the most severe patients: SaO2 < 85%, lactate concentrations >1.7 mmol/L and increased P50. That suggest for the authors, that a compromised respiratory function is associated with reduced Hb-O2 affinity (Bergamaschi et al., 2023).

#### Measurement of P50

In a methodology point of view, we have due to the retrospective analysis, calculated the P50 from the worst blood arterial sample at day 1 and day 3 making comparison with our study difficult. As reported by Böning et al. in their recent review (Böning et al., 2023), studies used different methodology: Ceruti et al. (2022) first used the Dash equation influenced by the Hill coefficient (Weiss, 1997) to calculate P50, based on the work carried out by Severinghaus (Severinghaus, 1979). This equation caused a decrease in P50 at extreme saturations (Böning et al., 2022). Ceruti therefore used the Hill equation corrected by Dash et al. (2016) following Böning’s comment (Böning et al., 2022). In the works from Vogel et al. (Vogel et al., 2020) and Valle et al. (Valle et al., 2022), the P50 was calculated by the Hill and Roche equation using data collected by the Cobas b221 blood analyser. In the Daniel et al. study (Daniel et al., 2020), P50 was measured automatically using the ‘TCS scientific’ analyser and the data was modified by a sigmoid function. There are many different methods for calculating P50, which makes it difficult to compare results as suggested by Böning et al. (2023). Furthermore, the formula used in our study made it possible to use all possible saturations (from 0% to 100%) and to remove confounding factors modifying the P50, such as pH, temperature and PaCO2 (Ceruti et al., 2022). This means that if the P50s differ, it is due to an additional factor and not to the confounding factors (Ceruti et al., 2022).

#### Etiologies of left shifting of the P50 and clinical relevance

Several hypotheses can explain the left shifting of the P50. The first, as suggested by Ceruti et al. (2022) and Valle et al. (2022) is that survivors have a greater capacity to compensate by hyperventilation, thereby inducing a respiratory alkalosis responsible for a leftward shift of the ODC. An animal study (Eaton et al., 1974) also showed that increasing the affinity of hemoglobin for O2 improved survival in hypoxic conditions.

A second hypothesis is that it could be due to alterations of the RBC rheology and/orbiochemistry. If these alterations were already observed in septic patients (Piagnerelli M et al., 2003; Reggiori et al., 2009; Piagnerelli M. et al., 2009), results in patients with COVID-19 were conflictual. Our group did not observed alterations of deformability in RBCs from patients with COVID-19 related ARDS (Piagnerelli et al., 2022) during the seven first day of ICU admission. That probably contribute to the adaptation of the microcirculation (increased red blood cells availability by vasodilatation of the microcirculation) observed in COVID-19 related ARDS by Favaron et al. (Favaron et al., 2021) like in hypoxemic conditions observed in healthy volunteers after exposure to high altitude (>5,000 m) (D’Alessandro et al., 2016). These authors observed a competitive binding of deoxyhemoglobin and glycolytic enzymes to the N-terminal cytosolic domain of Band 3. These modifications promote the accumulation of 2,3 DPG, stabilizing the deoxygenated state of hemoglobin, and cytolosic acidification, triggering O2 off-loading through the Bohr effect. This group observed the same alterations in the metabolism of RBCs from patients with COVID-19 with an increased levels of glycolytic intermediates, accompanied by oxidation and fragmentation of ankyrin, spectrin beta, and the N-terminal cytolosic domain of Band 3 (Thomas et al., 2020). The increased concentration of 2,3 DPG in RBCs from COVID-19 patients was recently reported by Bertilacchi et al., and if is the only cause induces a right shift of the ODC (Bertilacchi et al., 2024).

Other hypotheses could be possible to explain the left shifting of the ODC: the lower viral load in mild COVID-19 patients, or the increase in methemoglobin and carboxyhemoglobin levels in COVID-19 positive patients (Böning et al., 2023). The higher temperature in intubated COVID-19 patients contribute to a deviation of the curve to the right with no direct link to COVID-19 infection (Tobin et al., 2020). All these elements are normally minimized by the Hill formula corrected by Dash et al. (14,15). With regard to silent hypoxemia in COVID-19 patients, it has been reported that they do not present with dyspnea despite very marked hypoxemia. The danger of this is that patients have no symptoms and then suddenly deteriorate without warning (Tobin et al., 2020). Dyspnea is subjective, but the risk of respiratory decompensation can be assessed by increasing the respiratory frequency (Demoule et al., 2024). In this study, dyspnea was not recorded but the respiratory frequency was comparable between the three groups. This is measured by a sensor on the thorax, so it is possible that there is a bias.

The first hypothesis is that COVID-19 binds to the angiotensin 2-converting enzyme receptor in the brain and carotid arteries, which may cause a reduction in central dyspnea (Vogel et al., 2020; Villadiego et al., 2020). Böning et al. (Böning et al., 2021) explain this phenomenon by hypoxemia without hypercapnia, as the respiratory center is particularly sensitive to hypercapnia. Hypoxemia plays a role in dyspnea only when the PaO2 is below 40 mmHg during isocapnia (Dhont et al., 2020; Tobin et al., 2020). If we compare the results between non-intubated COVID-19 patients and non-COVID-19 patients, this statement is true, as the PaCO2 is lower in COVID-19 patients. This could contribute to this silent hypoxemia. On the other hand, PaCO2 is higher in intubated COVID-19 patients than in non-COVID-19 patients. This phenomenon is probably linked to the tolerance of hypercapnia during intubation in order to avoid the harmful effects of this (Bergamaschi et al., 2023).

#### Strengths and weaknesses of the study

Our study has the advantage of having been carried out on a large number of patients, divided into three groups, with a non-COVID-19 comparison group. P50 was measured at two different times during ICU stay, and the P50 formula was corrected to minimize bias. One of the limitations of this work is the corrected P50, which remains a mathematical measure with potential biases despite the correction. There is also no measurement of 2,3-DPG concentrations. With regard to the evolution of P50, an additional measurement would probably have been useful at the end of ICU stay to see if there was a correlation with mortality. Another weakness of this study is that P50 was measured on the first day in the ICU and not on the first day of hospital admission, so it is possible that P50 was different on admission to hospital.

In conclusion, this study found that there was a left shifting in ODC in ICU patients with COVID-19 related ARDS, especially in non-intubated patients, compared with non-COVID-19 patients. This deviation increases the third day only in non-intubated COVID-19 related ARDS and was not related to the outcome. The hypothesis of a direct link between COVID-19 and hemoglobin could explain this phenomenon, but the precise pathophysiological cause remains unknown.

## Data Availability

The raw data supporting the conclusions of this article will be made available by the authors, without undue reservation.
